# Visible Light Enhancement of Biocarbon Quantum-Dot-Decorated TiO_2_ for Naphthalene Removal

**DOI:** 10.3390/molecules29112708

**Published:** 2024-06-06

**Authors:** Yunteng Chen, Chunxian Hong, Qiang Xu, Haihong Zheng, Chao Wang, Hongshun Lu, Shuai Zhang, Mingming Du, Ganning Zeng

**Affiliations:** 1 Shaoxing Communications Investment Group Co., Ltd., Shaoxing 312099, China; 2 College of Environment, Zhejiang University of Technology, Hangzhou 310014, China; 3 College of Chemical Engineering, Zhejiang University of Technology, Hangzhou 310014, China; 4 China Construction Third Engineering Shanghai Co., Ltd., Shanghai 200082, China; 5 Hangzhou Construction Quality and Safety Supervision Station, Hangzhou 310012, China

**Keywords:** carbon quantum dots, TiO_2_, photodegradation, naphthalene

## Abstract

In this study, carbon-quantum-dot (CQD)-decorated TiO_2_ was prepared using an ultrasonic doping method and applied in the photocatalytic degradation of naphthalene under sunlight irradiation. The CQDs were synthesized from a typical macroalgae via diluted sulfuric acid pretreatment and hydrothermal synthesis using an optimal design, i.e., 3 wt% and 200 °C, respectively. The CQD/TiO_2_ composite remarkably enhanced the photocatalytic activity. The degradation of naphthalene under a visible light environment indicated that there is a synergistic mechanism between the CQDs and TiO_2_, in which the generation of reactive oxygen species is significantly triggered; in addition, the N that originated from the macroalgae accelerated the photocatalytic efficiency. Kinetic analysis showed that the photocatalytic behavior of the CQD/TiO_2_ composite followed a pseudo-first-order equation. Consequently, our combined experimental approach not only provides a facile pretreatment process for bio-CQDs synthesis, but also delivers a suitable TiO_2_ photocatalyst for the visible environment along with critical insights into the development of harmful macroalgae resources.

## 1. Introduction

Polycyclic aromatic hydrocarbons (PAHs) have teratogenic, carcinogenic, and mutagenic effects that likely threaten human health via biocommunication or the amplification of the food chain [1]. Among the diverse remediation technologies that can be applied to aquatic PAHs, TiO_2_ photocatalysis has garnered attention in recent years due its good photochemical stability, high catalytic efficiency, and strong capacity for oxidation [2,3]. However, due to its wide band gap and high photo-generated electron–hole pair recombination rate [4], unmodified TiO_2_ is often less active in the visible region and exhibits low carrier utilization; these factors greatly limit its widespread application.

Many research endeavors have been dedicated to developing modified TiO_2_ photocatalyst systems in order to maximize the utilization of visible light [5]; these carbon doping technologies have been found to help overcome some of the intrinsic shortcomings of TiO_2_, such as its low absorption and utilization of light, and to easily recombine photogenerated electrons and photogenerated holes [6]. Compared with other technologies utilized for the doping of carbon materials, CQDs are particularly attractive in this field due to their capacity for electron transfer, good photoluminescence, up-conversion fluorescence performance, and facile tunability [7,8]. However, the top-down method utilized for the preparation of CQDs still has certain limitations; for example, the carbon precursors are usually limited to materials with a large area of sp2 hybridization. In comparison, the bottom-up synthesis method employed for the preparation of CQDs has been widely used due to its simplicity and practicability [9,10]. It is worth noting that the development of simple, low-consumption, and gentle synthetic methods for the preparation of fluorescent CQDs and the use of readily available, inexpensive, and environmentally friendly natural resources as green carbon precursors has become an active research area. Biomass, especially renewable biomass materials, has been widely used as a precursor for the preparation of CQDs, showing good photoluminescence properties, low toxicity, and good biocompatibility [11,12]. However, its practical application is hindered by its low yield, low fluorescence intensity, and controllability, which are affected by its complex biological composition. Therefore, the preparation of CQDs from biomass, especially waste biomass, has become a research hotspot in recent years. With this in mind, a fast-growing macroalgae, i.e., Sargassum horneri (S.H.), whose burst is defined as golden tide, [13] was used as a CQD precursor.

On the other hand, the synergistic effect of CQD/TiO_2_ has been proven to effectively inhibit the recombination of electrons and holes and improve photocatalytic efficiency [14]. In CQD/TiO_2_ composites, CQDs are bonded to TiO_2_ by Ti-O-C bonding, and some electrons migrate to TiO_2_; this results in Ti^3+^ defects in the TiO_2_ matrix and a positive charge on the surface of the CQDs. Consequently, the presence of Ti^3+^ promotes the adsorption of O_2_^−^ on the photocatalyst surface [15]. Simultaneously, the excited electrons within the composites transfer from the valence band (VB) of the CQDs to the conduction band (CB) of TiO_2_ [16]. Sequentially, the electrons of CB in TiO_2_ react with O_2_ to form·O_2_^−^, and the holes on the surface of the CQDs further oxidize H_2_O or OH^−^ to form ·OH free radicals [17]; these two free radicals play an important role in the degradation of contaminants. As an important electron reservoir, CQDs can collect and store photogenerated electrons from the CB of TiO_2_, thereby hindering the recombination of electron–hole pairs and further promoting photocatalytic activity. Regarding TiO_2_, its light response generally ranges within the ultraviolet light region, and it is excited with difficulty by light sources larger than 420 nm because of the wide band gap. Meanwhile, CQDs can effectively up-convert visible light that has a wavelength greater than 420 nm into ultraviolet and near-ultraviolet light with a range of 350–550 nm [18,19]. Under light irradiation, the photogenerated electrons trapped on CQDs can further reduce the absorbed O_2_ to reactive ·O_2_^−^, the generation of which may depend on the separation efficiency of the photogenerated carriers and the number of photogenerated electrons captured by the CQDs. In particular, due to their graphite-like electronic structure and functional groups, CQDs can promote the adsorption of organic compounds by the complex, improve the area of contact with the target contaminants, and further carry out the photodegradation process. It is worth mentioning that the measurement of the transport band gap of the CQDs and TiO_2_ is also crucial, as the optical properties, the separation, and the migration of electrons in photocatalysts are the key factors that determine the photocatalytic activity [20,21,22].

The surface of biomass CQDs is rich in oxygen-containing functional groups [23,24,25]; they are therefore able to combine with semiconductors such as TiO_2_ to form potential photocatalysts. In recent years, algae and their derived carbon materials have been found to act as co-catalysts when coupled with TiO_2_ [26,27,28]. However, CQD/TiO_2_ photocatalysts prepared using biomass are also affected by their complex biological compositions. A suitable pretreatment method would destroy the cellulose of the natural plant structure and increase the specific surface area and porosity of the raw materials; therefore, the economic and facile pretreatment of biomass for sequential composite synthesis is still crucial and challenging. In order to improve the yield of carbon quantum dots, dilute acid can be used in the pretreatment of biomass, showing the ability to perform cellulose hydrolysis under relatively wild reaction conditions [29,30]. Therefore, the steps of our reaction are defined as follows: pretreatment with dilute acid, the hydrothermal preparation of CQDs, and the synthesis of CQD/TiO_2_ via ultrasonic dipping. Furthermore, the potential of CQD/TiO_2_ as a photocatalyst was investigated in the aquatic degradation of naphthalene under visible light. The aims of this study were to build a facile pretreatment process for bio-CQDs synthesis, to obtain a suitable method for the bio-CQD-decorated TiO_2_ catalyst suitable for a visible environment, and to find a potential industrial application for macroalgae.

## 2. Results

### 2.1. Effect of Dilute Sulfuric Acid Pretreatment on CQDs

The pretreatment with dilute acid was performed to hydrolyze and destroy the natural plant structure of the cellulose and simultaneously increase the specific surface area and porosity of the S.H. [31] as well as the yield of CQDs. This method is more convenient compared with both the traditional method using concentrated acid, from the viewpoint of contamination, or the method using ultra-low-dilute acid, from the viewpoint of energy demand; this is due to the high temperature and pressure that this method requires. Based on the results, an optimized design was chosen, i.e., 3.0% H_2_SO_4_ and a hydrothermal condition of 200 °C; with this, there was an almost 10-fold increase in the CQD yield, rising from 2.3% to 18.9% (Figures S1 and S2 in the Supplementary Materials). Regarding the synthesis of the CQD/TiO_2_ nanocomposite, 0.40 g of TiO_2_ and 1–10 mL of the CQDs(L) (liquid form, abbreviated as L), in which 1 mL of CQDs(L) corresponded to 0.012 g of CQDs(S), were dispersed into 30 mL of deionized water and processed using ultrasonic irradiation for 30 min. The suspension was dried at 60 °C for 24 h to achieve a CQD/TiO_2_ composite.

### 2.2. Characterization of CQDs and CQD/TiO_2_

**Figure 1:**
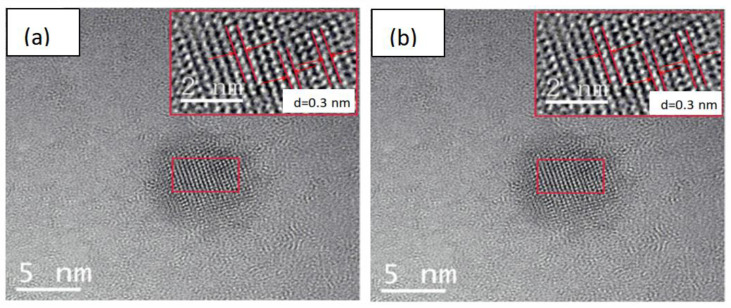
TEM image of (**a**) CQDs and (**b**) CQD/TiO_2_.

TEM analysis indicated that the CQDs presented an approximately spherical nano-morphological distribution with a nanometer size. The CQDs had a good in-plane lattice with a spacing less than 0.3 nm (Figure 1a), which is close to the (101) plane of graphite. The CQD/TiO_2_ had a good crystal plane spacing that was similar to that of TiO_2_, and the diameter of the CQDs had no obvious effect on the morphology of the TiO_2_. This indicates that there was a good binding structure between the CQDs and TiO_2_. Meanwhile, after embedding CQDs into TiO_2_, the lattice spacing of the CQD/TiO_2_ composite was determined to be more than 0.3 nm (Figure 1b).

### 2.3. UV–Vis Analysis of CQD/TiO_2_

**Figure 2:**
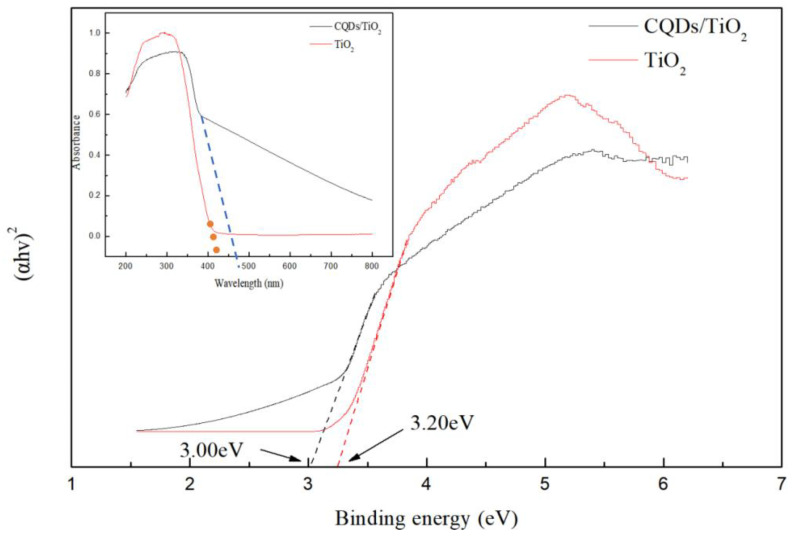
UV–Vis DRS results of CQD/TiO_2_ composite and TiO_2_.

In the ultraviolet wave band, the light absorption of CQD/TiO_2_ is less than that of TiO_2_, but in the visible light region, the light absorption of the CQD/TiO_2_ composite material is greatly enhanced compared with that of TiO_2_ (Figure 2). Because the CQDs exhibit a higher LUMO (lowest unoccupied molecular orbital) energy level than the TiO_2_, the electrons excited by visible light can be easily transferred from the LUMO energy level to the CB of TiO_2_, and the absorption intensity of the CQDTiO_2_ is much higher than that of TiO_2_ in the visible region [32]. The threshold wavelengths (λg) of the absorption spectra of simple TiO_2_ are obviously lower than those of the CQD/TiO_2_ composite, which is due to the near-infrared absorption characteristics of the CQDs [33] (Figure S3 in the Supplementary Materials). The light absorption red-shift and range broadening significantly indicated the promotion of the photocatalytic performance [34]. Further calculations also showed that the binding energy of the CQD/TiO_2_ (3.00 eV) was lower than that of TiO_2_ (3.20 eV), which means that more excited electrons transferred from the VB to the CB; this led to the promotion of the photo-absorption of the composite. That is to say, the red-shift performance of CQDs and their tight combination with TiO_2_ can effectively promote the red-shift of the absorption wavelength and effectively inhibit the recombination of electrons and holes. The band gap of CQDs/TiO_2_ is reduced relative to that of pure TiO_2_, and the interfacial interaction between CQDs and TiO_2_ leads to the redistribution or rearrangement of electron energy levels [35]. Especially, the biomass origin of CQDs results in a significant number of surface traps for electrons or holes, influencing the band structure of the composites. Therefore, surface modifications may further be considered to adjust the band gap width.

### 2.4. FTIR Analysis of CQD/TiO_2_

**Figure 3:**
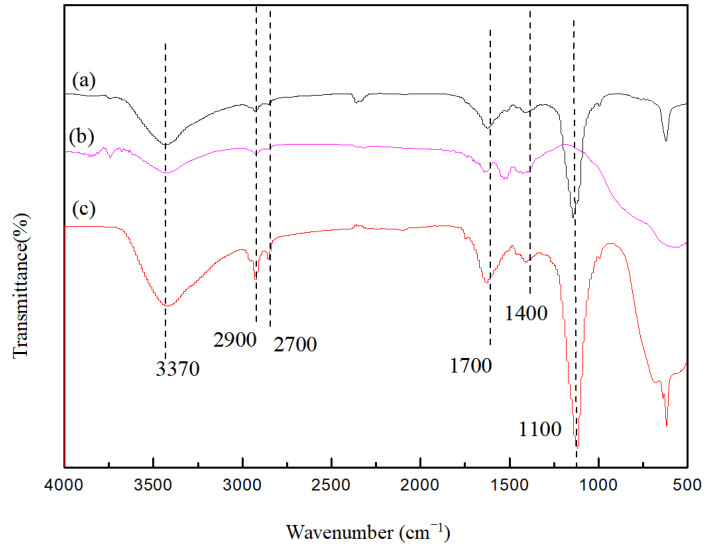
FTIR spectra of different materials: (**a**) CQDs (**b**) TiO_2_ (**c**) CQD/TiO_2_.

The FTIR spectra of different materials are illustrated in Figure 3. In the FTIR spectra, the broad and strong absorption band peak at 3370 cm^−1^ is assigned to the -OH stretching vibration, and the absorption peaks at 2900 cm^−1^ and 1700 cm^−1^ are the stretching vibration peaks of C-H and C=O, respectively. The conjugate characteristic absorption peak produced by C=C appears near 1400 cm^−1^, and the absorption peak at 650–900 cm^−1^ may be the benzene ring hydrogen absorption peaks of in-plane, out-of-plane, and benzene ring skeleton-bending vibration. The FTIR analysis indicates that carbonaceous groups were introduced on the surface of the TiO_2_, thus confirming the formation of the CQD/TiO_2_ composite. Meanwhile, the combination of CQDs enhances the absorption intensity of the Ti-O bond at 1100 cm^−1^, and this peak is the characteristic absorption peak of the Ti-O bond. In addition, the CQD/TiO_2_ shows a strong capacity for absorption when the wave number is lower than 1000 cm^−1^, which may be related to the increased distance between atoms or lattices and the characteristic absorption peaks of Ti-O-C.

### 2.5. XPS Analysis of CQD/TiO_2_

**Figure 4:**
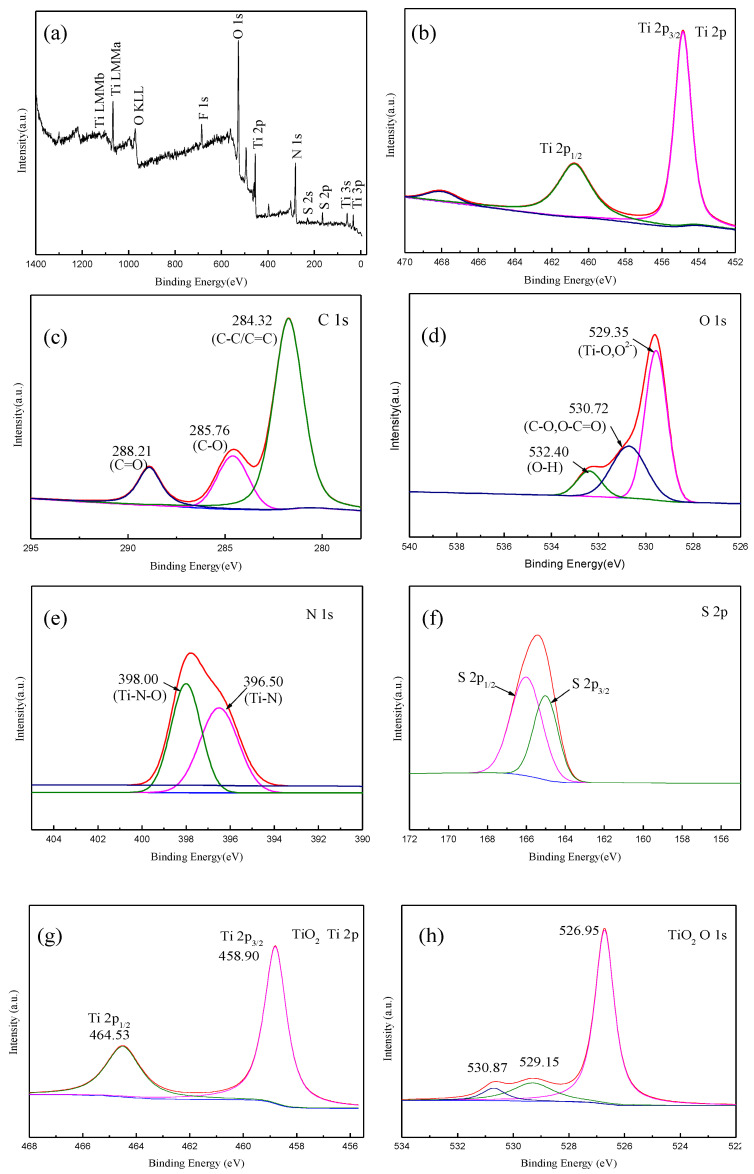
XPS spectra of CQD/TiO_2_ composite materials: (**a**) full spectrum; (**b**) Ti 2p spectrum; (**c**) C 1s spectrum; (**d**) O 1s spectrum; (**e**) N 1s spectrum; (**f**) S 2p spectrum; (**g**) Ti 2p spectrum of TiO_2_; (**h**) O 1s spectrum of TiO_2_.

To explore the surface chemical composition and related valence state of the CQD/TiO_2_, the XPS full spectrum is given in Figure 4. Figure 4a shows that the composite contains the elements Ti, O, C, S, and N. The Ti 2p spectrum exhibited two peaks at 454.72 eV and 460.53 eV, which corresponded to Ti 2p3/2 and Ti 2p1/2, respectively; these are assigned to Ti^4+^ 2p peaks [36,37]. The binding energy of Ti 2p is shifted from the standard value of TiO_2_, indicating that there is a new binding structure in the CQD/TiO_2_ composite. The binding energy of Ti 2p3/2 is lower than the standard value of 458.20 eV, indicating that the existence of CQDs makes the electron-binding energy smaller and increases the electron density of the TiO_2_. Figure 4c shows the C 1s spectrum. The peaks of the C 1s spectrum at 284.32 eV, 285.76 eV, and 288.21 eV are attributed to the C-C/C=C, C-O, and C=O bonds, respectively. Figure 4d shows that the absorption peaks of 529.35 eV, 530.72 eV, and 532.40 eV in the O 1s spectrum are the characteristic peaks of the Ti-O/·O_2_, C=O, and C-O groups, indicating the composition of the surface of the CQD/TiO_2_ composite material. The peak at 529.35 eV was attributed to the oxygen in the crystal lattice (Ti-O/O_2_), and the other two peaks at 530.72 eV and 532.40 eV were attributed to the C=O and C-O groups, indicating that a hybrid might have been formed in the CQD/TiO_2_ by a Ti-O-C bond. Figure 4e,f show the spectral lines of N 1s and S 2p. The absorption peaks of the N 1s spectrum at 396.50 eV and 398.00 eV are Ti-N and Ti-N-O bonds, respectively. The binding energies of S 2p 3/2 and S 2p1/2 are located at 165.3 eV and 166.8 eV, respectively, mainly due to the S-C-S bond. N and S originated from the biomass, and the latter also was enhanced by the inclusion of the dilute sulfuric acid pretreatment. The doping of N and S also promoted the performance, thus indicating the advantages of biomass self-assembly [38]. In summary, the XPS analysis confirmed the presence of CQDs and their TiO_2_ counterparts in the structure of the composites.

### 2.6. Photocatalytic Performance of CQD/TiO_2_ on Naphthalene Removal

**Figure 5:**
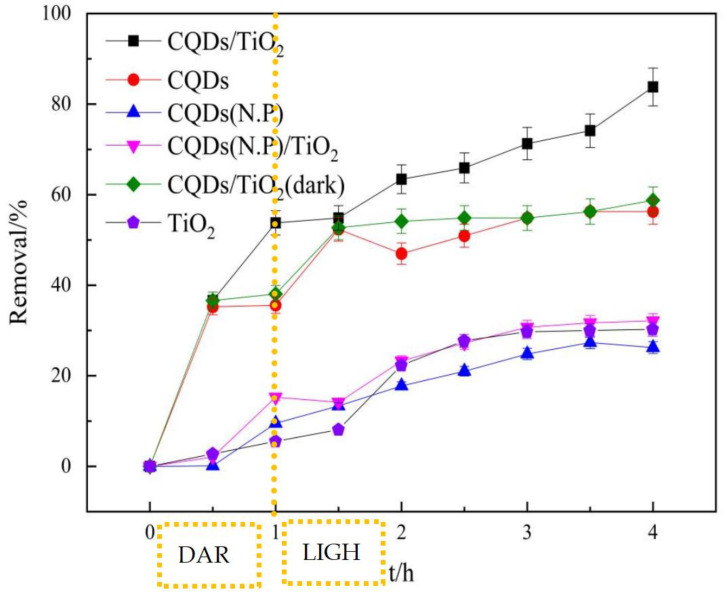
Effect of different materials on photocatalytic reaction of naphthalene. (N.P. means without pretreatment using dilute sulfuric acid).

In order to certify the photocatalytic intensification of CQDs and the dilute acid treatment, the degradation of naphthalene was performed using different materials, as shown in Figure 5. The initial environment parameters were as follows: the naphthalene concentration was 40 mg·L^−1^ and the volume was 500 mL; the CQD/TiO_2_ was synthesized using a mixture with a ratio of 10 mL of CQDs to 0.40 g of TiO_2_; and 0.03 g of solid material was used. It can be seen that the photocatalytic effect of CQD/TiO_2_ is the best among all the materials, indicating that the doping of CQDs is beneficial to the photocatalytic performance. The sulfuric acid pretreatment helps the CQDs to promote adsorption, thus leading to their tight combination with TiO_2_ and promoting the photocatalytic performance of CQD/TiO_2_.

**Figure 6:**
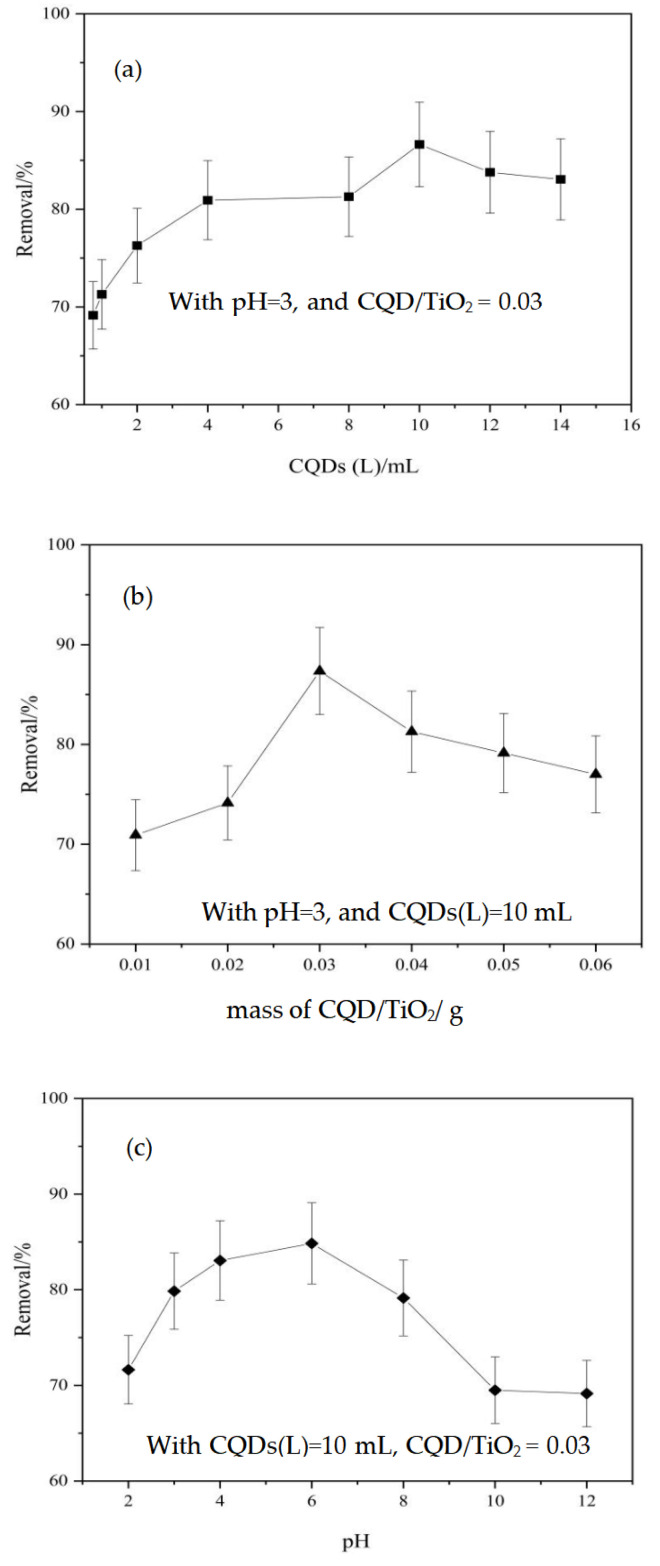
Effect of photocatalytic degradation of CQD/TiO_2_ on naphthalene. (**a**) CQDs; (**b**) mass of CQDS/TiO_2;_ (**c**) pH.

It is worth mentioning that adsorption continued to play an important role regardless of whether the dark or light condition was used; this is because the CQDs continued to mix with biomass carbon. In particular, with the increase in the carbon content, the adsorption of biomass carbon becomes crucial to the composite; meanwhile, the increase in the proportion of TiO_2_ used makes the photocatalytic effect more obvious [31]. It could be found that the ratio of CQDs in the CQD/TiO_2_ composite leads to variations in the removal efficiency, and an overdose of CQDs in TiO_2_ may cause particle aggregation and pore blocking and thus reduce the photocatalytic efficiency (Figure 6a). When the loading amount of CQDs (L) is below 1 mL, the photocatalytic efficiency of CQDs/TiO_2_ in naphthalene solution increases with the decrease in CQDs. This could be due to CQDs obstructing some TiO_2_-indicated holes. As CQDs decrease, fewer holes are blocked, allowing TiO_2_ to dominate the solution’s photocatalytic efficiency. As the CQD content in the composites increased, the photocatalytic efficiency of the naphthalene solution initially rose, reaching 86.63% at a loading of 10 mL, before slightly declining with further CQD addition. The synergistic effect of CQDs and TiO_2_ on electron–hole pairs likely optimizes the photocatalytic efficiency of the composites for naphthalene solution. Further increasing CQD loading may lead to agglomeration and adherence to the titanium dioxide’s surface, impeding titanium dioxide’s light utilization and consequently diminishing the photocatalytic efficiency of the CQDs/TiO_2_ in naphthalene solution. This overdose effect was most obvious with the composite mass; the photocatalytic efficiency of the naphthalene solution was the best with the mass of CQD/TiO_2_ achieving 0.03 g (Figure 6b). Obviously, suitable content of the constituent components can always optimize the photocatalytic performance. As a matter of fact, three internal approaches, i.e., quantitative effect, compositional engineering, and size quantization, have been simultaneously yet unintentionally exerted in bio-CQDs doping TiO_2_ [39,40]. Regarding the pH effect, the composite catalyst had a good catalytic effect on naphthalene around pH = 6 (Figure 6c), which was greatly affected by TiO_2_; this increased the acid–basic balance to pHzpc ~6.39 [41,42]. When the pH value of the reaction system is less than pHzpc, the increase in H^+^ in the solution will cause Ti-OH^2+^ generation; this is suitable for attracting photogenerated electrons and thereby effectively reducing the recombination rate of photogenerated electrons and holes. The best dose of CQD/TiO_2_ could be a result of the balance between two aspects, i.e., the active sites involved in the reaction and the capacity of the receiving photons.

## 3. Discussion

### 3.1. Photocatalytic Mechanism Analysis of CQD/TiO_2_ on Naphthalene Removal

**Figure 7:**
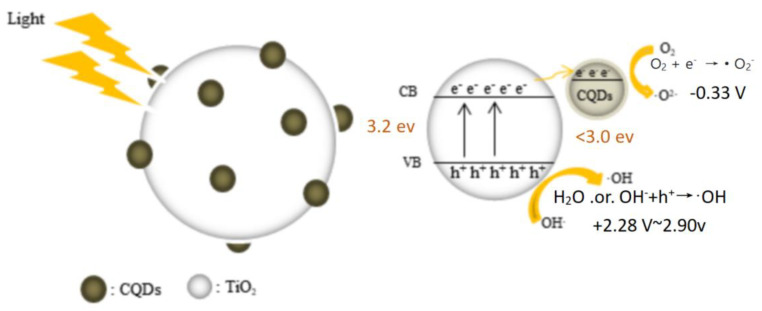
Synergy mechanism of CQD/TiO_2_.

In this work, the results of FTIR, ultraviolet–visible spectroscopy, and fluorescence spectroscopy showed that there were abundant functional groups on the surface of the CQD/TiO_2_ and that the absorption peaks at wavenumbers lower than 1000 cm^−1^ were the characteristic absorption peaks of Ti-O-C. The band gap of CQD/TiO_2_ was reduced compared with that of TiO_2_, and the absorption band shifted to visible light; meanwhile, the interfacial interaction between the CQDs and TiO_2_ may lead to the redistribution or rearrangement of electron energy levels [35]. Furthermore, the XPS results also indicated the variation in the surface elements of the composites, and the results showed that the electron density of the outer layer of Ti increased and the electron-binding energy decreased. In addition, the Ti 2p line showed that CQDs and TiO_2_ were bonded through Ti-O-C bonds. The mechanism is illustrated in Figure 7. The binding energy of TiO_2_ was given according to our determination, whereas the binding energy of the CQDs/TiO_2_ was found to be 3.0 eV. Herein, we refer to the binding energy of CQDs as being less than 3.0 eV, which aligns with the bio-CQD results published in [43]. The redox potentials of H_2_O/·OH and OH^−^/·OH are +2.28 V, +2.9 V, respectively, and the potential of O_2_/O_2_^−^ is −0.33 V [44,45,46]. However, it is crucial to acknowledge that reactive species and their exact potentials may be affected by the local environment. The photo-induced electron transfer and redox properties of CQDs improve the separation time of electron–hole pairs of TiO_2_, preventing the recombination of electron holes. This provides more time for the contaminants to diffuse to the reactive site, which accelerates the photocatalytic efficiency. CQDs can be used as an electron reservoir during photocatalysis, and the excited electrons of CQD/TiO_2_ can be transferred from the valence band (VB) of the CQDs to the conduction band (CB) of the TiO_2_, thereby hindering the recombination of electron–hole pairs and further promoting the photocatalytic activity. The generation of reactive oxygen species (ROS) can be triggered by low-power visible light irradiation [47]; i.e., the electrons of CB in TiO_2_ react with O_2_ to form ·O_2_^−^. The photogenerated electrons trapped on CQDs can further capture the absorbed O_2_ to reactive ·O_2_^−^, and the holes on the surface of CQDs oxidize H_2_O to form ·OH [48,49]. This provides more time for the contaminants to diffuse to the reactive site, which accelerates the photocatalytic efficiency. In addition, the N originating from the biomass makes TiO_2_ exhibit p-type conductive properties [50,51], which makes it easier for electrons to transfer to CQDs and improves the photocatalytic efficiency. In particular, the biomass origin of CQDs introduces a significant number of surface traps for electrons or holes, affecting the band structure of the composites. Consequently, further consideration of surface modifications may be necessary to fine-tune the band gap width.

**Figure 8:**
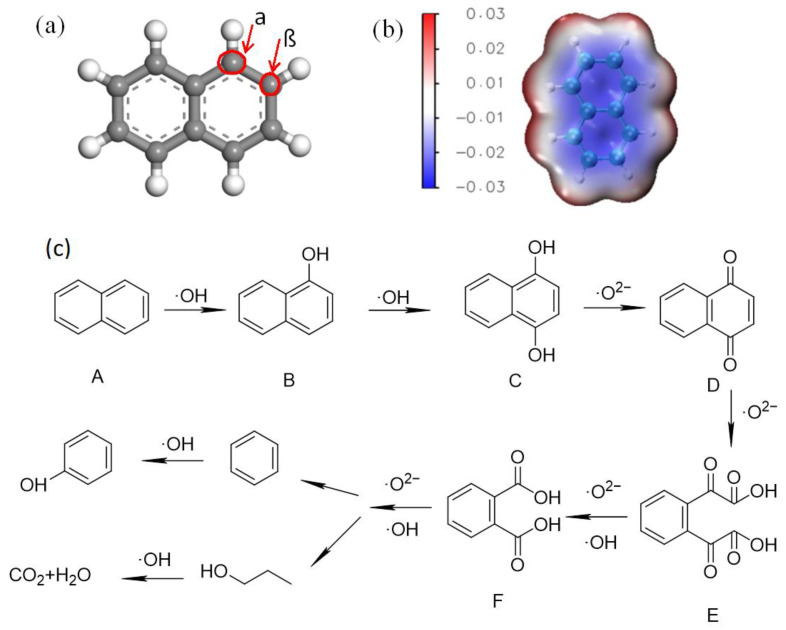
Degradation of naphthalene with CQD/TiO_2_: (**a**) the chemical structure of naphthalene; (**b**) electrostatic potential (ESP) distribution of naphthalene; (**c**) degradation pathway map of naphthalene, in which products A to F are Naphthalene, Hydroxyl derivatives, naphthol, naphthoquinone, aldehyde compound, Carboxyl compounds, respectively.

Regarding the degradation of naphthalene, the existence of ·O_2_^−^ and ·OH enable the breaking or cleaving of the ions trapped on the CQDs; this leads to the further capture of absorbed O_2_ and reactive ·O_2_^−^ on the halobenzene ring structure. There are two types of hydrogen on the naphthalene structure, namely, α-H and β-H (Figure 8). Compared with β-H, α-H has a higher electron density and activity, which means that this site is more easily connected with free radicals. The surface of naphthalene molecules is positively charged, and the electrostatic attraction between atoms can promote the negatively charged ·OH attacks on H at the α position, causing the C at the α position to form hydroxyl derivatives and generate naphthol (products B and C). ·O_2_^−^ further oxidizes naphthol to form C=O double bonds, forming naphthoquinone (product D). Naphthoquinone is further attacked by ·OH, leading to the cleavage of C–C bonds to form aldehyde compound E. Due to free radical attack at C1 and C4, the resulting aldehyde group is easily oxidized to a carboxyl group, leading to the formation of the product F. These intermediates are further oxidized into smaller molecules and are eventually fully mineralized.

### 3.2. Kinetic Model Analysis of Naphthalene Removal by CQD/TiO_2_

The pseudo-first-order kinetic model, the second-order kinetic model, and the double exponential kinetic model were used to fit the photocatalysis of CQD/TiO_2_. The relevant parameters of the kinetic model are shown in Table 1, and the fitting plots can be found in Figure S4 (Supplementary Materials). The results indicate that the pseudo-first-order rate equation provides the best interpretation of the photocatalysis of naphthalene when the CQD/TiO_2_ is used. Meanwhile, the double exponential model provides the best fit for the CQDs, indicating that both physical and chemical processes are involved in the degradation of naphthalene using CQDs and that a change was experienced after combination with TiO_2_. Thus, naphthalene can diffuse thoroughly toward the reaction active site, which accelerates the photocatalytic reaction.

## 4. Materials and Methods

### 4.1. Materials

S.H. was collected from Wenzhou coastal area and washed with deionized water to remove salts and impurities on the surface before being dried in an electric blast oven for 24 h. The dried S.H. was then ground and sieved through an 80-mesh sieve. Reagents were acquired from commercial suppliers and used without any further processing or purification. Titanium dioxide (P25, Degussa, 99.5%) was procured from Sinopharm Chemical reagent Co., Ltd., and chromatographic pure naphthalene was purchased from Aladdin Reagent Company. Analytically pure acetic acid, sulfuric acid (H_2_SO_4_), hydrochloric acid (HCl), sodium hydroxide (NaOH), and dichloromethane were purchased from Lingfeng Chemical Reagent Co., Ltd. (Shanghai, China).

### 4.2. Preparation of CQDs and CQD/TiO_2_

Sulfuric acid at concentrations of 1.5 wt%, 3.0 wt%, and 4.0 wt% was used for dipping with S.H. Dilute sulfuric acid was dissolved using 50 mL of de-ionized water in a 100 mL beaker, and the solution was transferred to a 100 mL stainless steel reactor; it was then placed in a drying oven at a predetermined temperature for 3 h. The 3.0 wt% concentration demonstrated a higher yield of CQDs and was thus selected for subsequent processes (Figure S5 in the Supplementary Materials). The diluted acid pretreatment was implemented because it effectively decomposes hemicellulose and lignin from biomass, facilitating the synthesis of carbonaceous materials [52]. After the reaction, the CQD solution was cooled to room temperature. Then, centrifugation was performed 3 times at a speed of 10,000 rpm·min^−1^ to remove the large particles; this was followed by neutralization and separation using a 0.22 μm polyethersulfone membrane. Sequentially, the carbon quantum dot (CQD) solution was obtained and designated as CQDs(L) (L means liquid). The CQD solution was freeze-dried to obtain a solid powder of CQD; this was as designated as CQDs(S) (S means solid).

### 4.3. Characterization of CQDs and CQD/TiO_2_

The morphology, size, and crystal lattice of the materials were analyzed using transmission electron microscopy with a field emission gun (TEM, Tecnai G2 F30, FEI Company Eindhoven, Eindhoven, The Netherlands). In detail, the parameters were as follows: the excitation voltage was 300 kV, the line resolution was 0.1 nm, the point resolution was 0.2 nm, and the information resolution was 0.14 nm. A Fourier transform infrared spectrometer (FT-IR, Nicolet IS50, Thermo Fisher Scientific, Waltham, MA, USA) was used in the range of 400–4000 cm^−1^ with a resolution of 4 cm^−1^ to characterize the surface function groups of the composites. UV–Vis absorption measurements were carried out via UV–Vis diffuse reflectance spectrophotometry (DRS) (CARY 300, Agilent, Palo Alto, CA, USA), with scanning in the range of 200–800 nm. The obtained data were processed according to the Tauc plot method. The surface chemical composition and chemical status of the CQD/TiO_2_ were investigated using X-ray photoelectron spectroscopy (XPS, Kratos Axis-Ultra, Kratos, Manchester, UK). In this paper, Al/Mg was used as the radiation source. The test conditions were as follows: the excitation source was Al Kɑ (1487 eV), the target voltage was 15 kV, the current was 3 mA, the vacuum degree was 10^−7^ Pa, and the binding energy was corrected with C1s and a reference of 284.6 eV.

### 4.4. Photocatalytic Activity Measurements

The photocatalytic activity of the CQD/TiO_2_ composites was evaluated using naphthalene. The photodegradation experiments were performed by using a photochemical reactor (PhChem-III, Newbit Technology Co., Ltd., Beijing, China) equipped with a xenon arc lamp (500W, XE-JY500, Ushio, Inc., Tokyo, Japan) to simulate sunlight irradiation (Figure S6 in the Supplementary Materials). The luminous power can be controlled by adjusting the resistance of the power supply. In this experiment, the luminous power was controlled at 300 watts. The catalysts were added to 10 mL of naphthalene solution (40 mg L^−1^) and stirred for 60 min under dark conditions to ensure that adsorption–desorption equilibrium was reached before photocatalysis was performed. During the photocatalysis process, the concentrations of naphthalene were determined using gas chromatography (GC-112A, Yidian Analytical Instrument Co., Ltd., Shanghai, China), and the relative removal rate (R) of naphthalene was calculated based on triplicate experiments in an air-conditioned room to prevent heat effects. The detection conditions were as follows: the column used was the Agilent DB-5 capillary column (30 m × 0.32 mm × 0.25 μm); the detector used was a hydrogen flame ionization detector; the detector temperature was 280 °C; the inlet temperature was 250 °C; the column temperature was programmed to be maintained at 60 °C for 1 min, 4 °C·min^−1^ to 130 °C for 5 min, and then 20 °C·min^−1^ to 280 °C; the carrier gas used was high-purity nitrogen, with a flow rate of 2.5 mL·min^−1^; the hydrogen flow rate was 40 mL·min^−1^; and the air flow rate was 400 mL·min^−1^. The gas chromatography results were presented using the internal standard–standard curve method. The determination of naphthalene and its degradation products was based on gas chromatography–mass spectrometry [53] (GC-MS, Agilent 7000D, Agilent Technologies, Santa Clara, CA, USA) by employing the DB-624 capillary column (30 m × 0.32 mm, 1.8 μm) with helium as the carrier gas and a flow rate of 1.5 mL/min. Under these conditions, comprehensive qualitative analysis was conducted using an electron ionization source, and quantitative analysis was performed via ion external standard method. The injector temperature was set at 150 °C utilizing split injection with a split ratio of 5:1. The programmed temperature conditions were set as follows: an initial temperature of 40 °C held for 1 min, then increased to 190 °C at a rate of 8 °C/min, further increased to 200 °C at a rate of 5 °C/min, and held for 7 min. Afterwards, the total ion chromatogram (TIC) was generated and identified.

## 5. Conclusions

CQDs were prepared using a N,S-containing marine biomass and a facile hydrothermal method, and CQD/TiO_2_ composites were synthesized using an ultrasonic method. This work indicates that macroalgae can act as a good precursor for CQDs, whose adsorption and combination performance can be enhanced by dilute sulfuric acid pretreatment; this further enhances the synthesis of CQD/TiO_2_ and its photocatalytic capacity. The nanostructure of CQDs means that they compound well with TiO_2_, and thus the photocatalytic performance of TiO_2_ can be significantly promoted under this synergistic effect. Generally speaking, the red-shift performance of CQDs and their tight combination with TiO_2_ can be demonstrated by the red-shift of the threshold wavelength, a decrease in the binding energy, and the further promotion of electron transfer, which enable a better degradation capacity to be achieved under visible light. During the degradation process, CQDs can be used as electron reserves in photocatalysis, thereby promoting the separation efficiency of electron–hole pairs and further free radicals. They also possess the advantages engendered by biocarbon adsorption due to the intermolecular accumulation of π-π. Therefore, under simulated light irradiation, CQD/TiO_2_ exhibits an excellent photocatalytic performance, and the removal of naphthalene is significantly higher than that of simple TiO_2_. The photocatalytic efficiency of the composite material seems sensitive to pH, especially during direct recycle tests; however, weak acid treatments can maintain its stability (Figure S7 in the Supplementary Materials). The observed differences can be attributed to the CQDs’ solubility and their physical loss during the washing process. Additionally, alternations in the crystal change can also lead to a reduction in the crystal structure integrity, which indicates that a more meticulous acid modification should be involved in future work.

## Figures and Tables

**Table 1:** Kinetics parameters for the removal of naphthalene.

Kinetic Model	Parameters	Samples		
		CQD/TiO_2_	CQD/TiO_2_ (dark)	CQDs
Pseudo-first-order	*k*_1_ × 10^2^/(min^−1^)	0.603	0.008	0.104
	*R* ^2^	0.8968	0.8608	0.4932
Pseudo-second-order	*k*_2_ × 10^4^/(L·mg^−1^·min^−1^)	5.642	0.447	0.552
	*R* ^2^	0.7764	0.8480	0.5179
Double exponential	A_1_	−41.89	−29.56	−28.14
	A_2_	−41.89	−29.56	−28.14
	*k*_3_	0.0588	0.0214	0.1529
	*k*_4_	0.0588	0.0214	0.1529
	*R* ^2^	−0.4477	0.2531	0.9150

## Data Availability

Data are contained within the article and Supplementary Materials.

## Supplementary Materials

The following supporting information can be downloaded at: https://www.mdpi.com/article/10.3390/molecules29112708/s1. Figure S1, Fluorescence spectra of S.H. CQDs with different hydrothermal temperature. Figure S2, Fluorescence spectra of S.H. CQDs with H_2_SO_4_ pretreatment after 200 °C hydrothermal treatment. Figure S3, CQDs (a) UV–Vis spectrum and (b) fluorescence response spectrum. Figure S4, Kinetic model fitting curves. Figure S5, (a) *Sargassum Horneri*; (b) *Sargassum Horneri* powder; (c) CQDs under natural light irradiation; (d) CQDs under ultraviolet light irradiation. Figure S6, The photocatalytic reactor apparatus. Figure S7, Effect of cycle times on photocatalysis of naphthalene.
