# Supplementary material for: Visible Light Enhancement of Biocarbon Quantum-Dot-Decorated TiO2 for Naphthalene Removal
Source: Molecules. 2024 Jun 6;29(11):2708. doi: 10.3390/molecules29112708 (PMC11173786; doi:10.3390/molecules29112708)
Supplement: Supplementary file 1 [file molecules-29-02708-s001.zip › molecules-2982018-supplementary.pdf]

Supporting Information

Different hydrothermal conditions showed 200 ºC is the best hydrothermal temperature for CQDs synthesis (Figures S1 and S2). Further investigation showed that 3% H_2SO_4 is the best pretreatment method both from CQDs yield view or fluorescence , almost 10 times CQDs could be achieved compared with no H_2SO_4 addition (Figure S3 and Table S1).

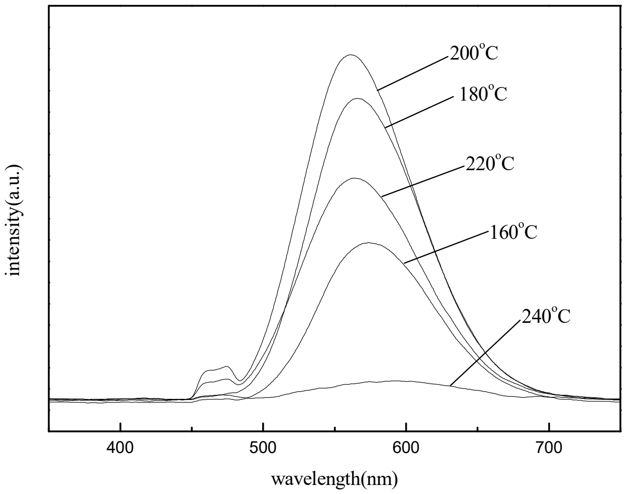

Figure S1. Fluorescence spectra of S.H. CQDs with different hydrothermal temperature

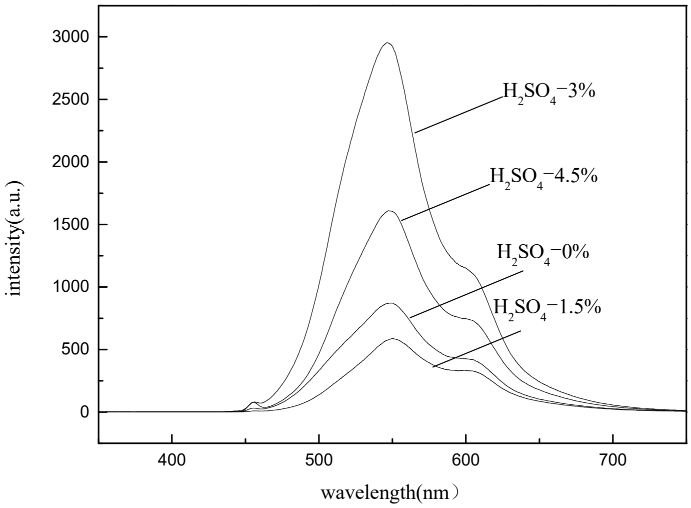

Figure S2. Fluorescence spectra of S.H. CQDs with H_2SO_4 pre-treatment after 200 °C hydrothermal treatment

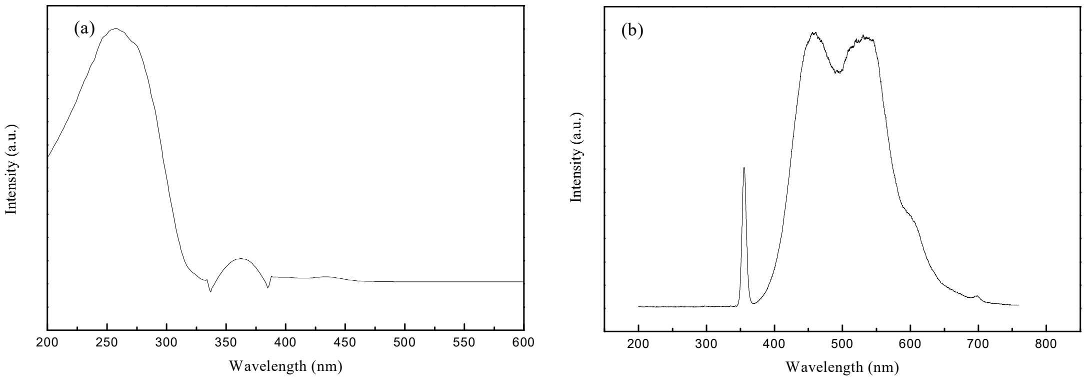

Figure S3. CQDs (a) UV-Vis spectrum and (b) fluorescence response spectrum

Table S1. pretreatment by diluted H_2SO_4 and corresponding S.H. CQDs yield

Pretreatment	CQDs yield
none	2.3%
1.5% H_2SO_4	18.0%
3.0%H_2SO_4	18.9%
4.5%H_2SO_4	13.3%

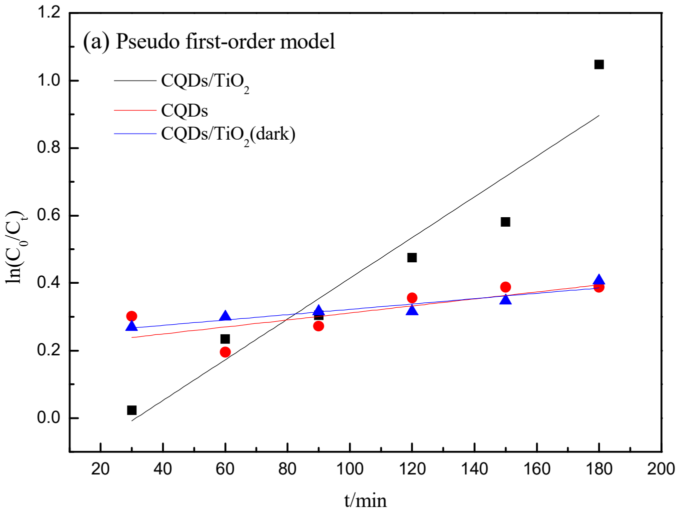

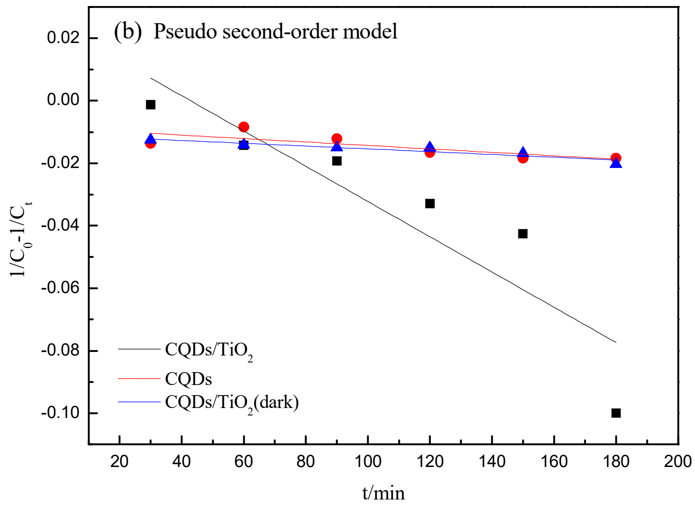

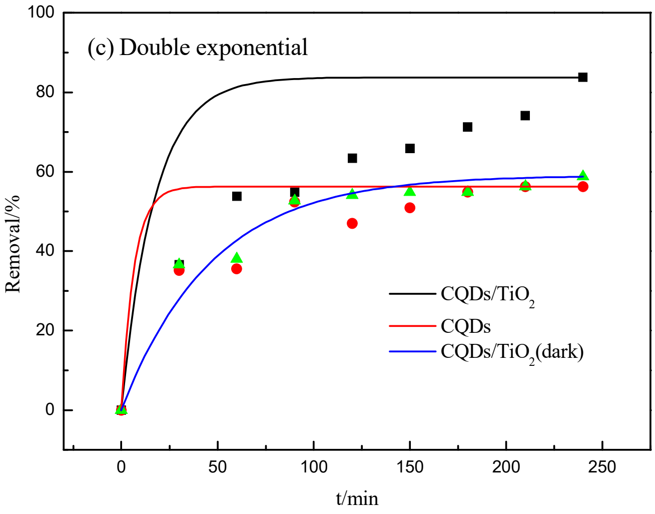

Figure S4. Kinetic model fitting curves

Figure S5 showed the initial S.H., the meshed S.H. powder and typical CQDs under natural light irradiation and under ultraviolet light irradiation. As can be seen from Figure S5, aqueous solution of CQDs was brown under natural light irradiation, and emitted a bright blue-green fluorescence at a wavelength of 360 nm under UV light irradiation.

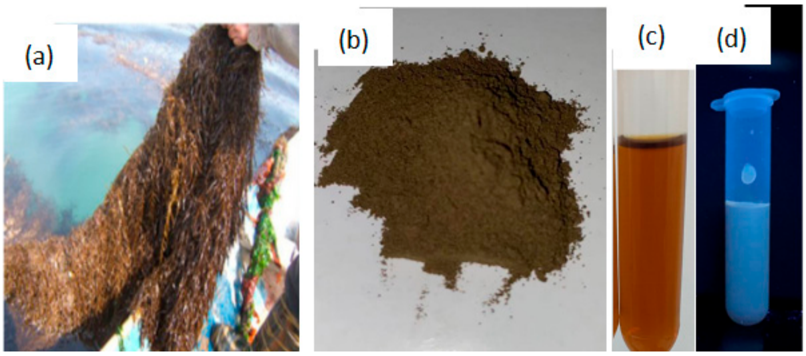

Figure S5. (a) *Sargassum Horneri*; (b) *Sargassum Horneri powder;* (c) CQDs under natural light irradiation; (d) CQDs under ultraviolet light irradiation

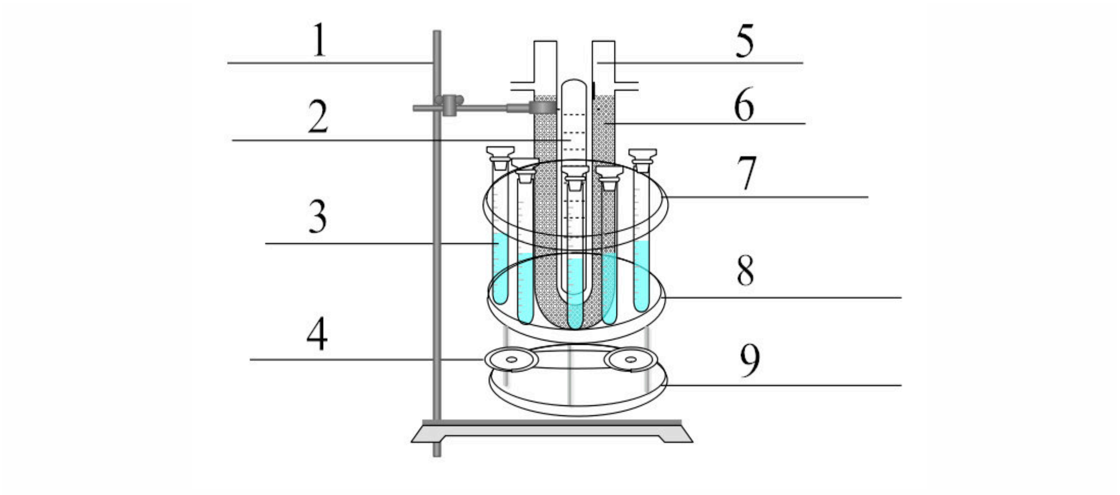

Figure S6. The image of apparatus of photocatalytic reactor

1.support frame; 2.modulator tube; 3.quartz reaction tube; 4.conveyor belt; 5.quartz cold trap; 6.cooling water; 7.top turntable; 8.bottom turntable; 9.support base

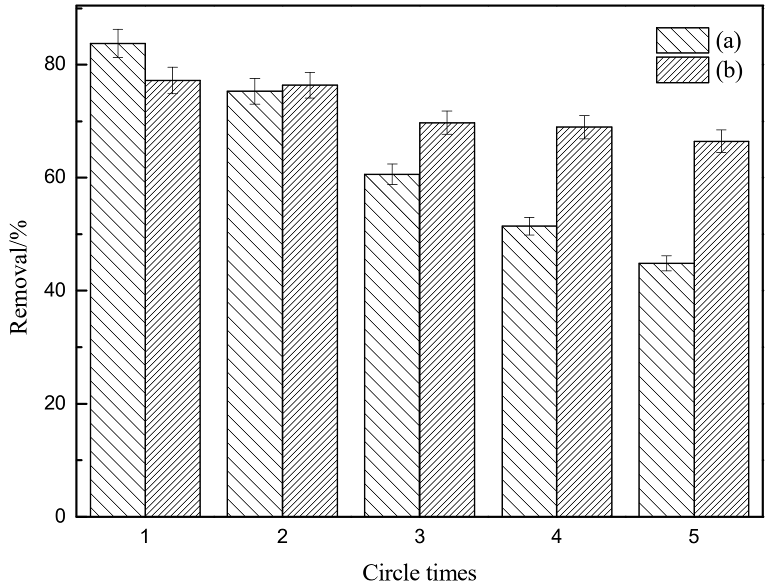

Figure S7. Effect of cycle times on photocatalytic on naphthalen Compound conditions (a) neutral (b) weak acid
